# Connecting two arrays: the emerging role of actin-microtubule cross-linking motor proteins

**DOI:** 10.3389/fpls.2015.00415

**Published:** 2015-06-02

**Authors:** René Schneider, Staffan Persson

**Affiliations:** ^1^Max-Planck-Institute for Molecular Plant Physiology, Potsdam-Golm, Germany; ^2^ARC Centre of Excellence in Plant Cell Walls, School of Botany, University of Melbourne, Parkville, VIC, Australia

**Keywords:** actin filaments, microtubules, kinesin-14, calponin homology domain, dynein

## Abstract

The cytoskeleton of plant cells, consisting of actin filaments (AFs) and microtubules (MTs), is a central structure for various intracellular processes, such as cell division, isotropic and polar growth, vesicle transport, cell shape, and morphogenesis. Pharmaceutical and genetic studies have provided indications for interdependent cross-talk between the cytoskeletal components. Recent live-cell imaging studies have cemented this notion, in particular when the cytoskeleton rearranges. However, the proteins that directly mediate this cross-talk have remained largely elusive. Recent data indicate that certain proteins can interact with both cytoskeletal arrays at the same time, and hence connecting them. In this review, we summarize the recent literature of the AF- and MT-interactors, mainly focusing on a plant-specific mediator of cytoskeletal cross-talk: the calponin homology (CH) domain-containing kinesin-14 motor proteins (KCHs).

## Introduction

All organisms possess intracellular filamentous structures, commonly summarized as the cytoskeleton, which are essential to many central processes, such as cell division, polar growth, and vesicular transport. In eukaryotes the cytoskeleton comprises of seven nm thick actin filaments (AFs), built from globular actin subunits, and 25 nm thick microtubules (MTs), built from α-/β-tubulin heterodimers. A third group, the intermediary filaments, assembled from a family of tetrameric protein subunits, is apparent in prokaryotes, yeast and animal cells but has so far not been identified in plants (Herrmann and Strelkov, 2011). In mammals, intermediary filaments—such as keratin that makes up hairs, nails and horns—give strength to the epidermal cell layers. In plants, the function of this missing exoskeletal structure is provided by a strong but flexible cell wall, comprised of 10–30 nm thick cellulose microfibrils. However, cellulose microfibrils are produced at the inner surface of the extracellular cell wall (McFarlane et al., 2014), and are not considered to be part of the plant cytoskeleton.

The term “cytoskeleton,” being coined long before electron and live-cell microscopy were established (Kotzloff, 1903), implies a somewhat misleading notion: instead of resembling rigid “bones,” AFs and MTs are highly dynamic structures that undergo constant switching between phases of polymerization (growing) and depolymerization (shrinking; Desai and Mitchison, 1998; Blanchoin et al., 2010). Even in differentiated cells the cytoskeleton is constantly reforming with growth rates of several thousands of nanometers per second for AFs, and some hundreds of nanometers per second for MTs. While the AFs and MTs are considered to be in a dynamic equilibrium, they can nonetheless withstand and generate considerable amounts of force (Dogterom et al., 2005; Footer et al., 2007).

The growth of a plant cell is tightly associated with major re-arrangements of AFs and MTs. Currently, there is little consensus on how these re-arrangements are achieved and if cross-talk between AFs and MTs is necessary. One striking example providing insight into these questions was recently presented by Lindeboom et al. (2013), who found that the reception of blue light caused a rapid 90° reorientation of the transverse MT array in growing hypocotyl cells—notably, AFs did not participate actively in this process. In this example, phototropin light receptors mediated the activation of KATANIN, a MT severing protein complex, which bound to cortical MT crossovers where it catalyzed the severing of the newly formed MTs. This led to the generation of new MTs that were oriented largely longitudinally to the growth axis of the cell, and thus perpendicular to the pre-existing MT array. Since the newly formed MTs repeatedly went across the pre-existing MTs, the KATANIN activity rapidly amplified the longitudinal array. For this mechanism to work at least one MT needs to initially deviate from the transverse orientation of the existing MT array. In this regard, the authors reported that some MTs obtained an orientation different from the MT array through curved growth trajectories. Although this might happen at random, it could also be envisioned that another “directional lattice or vectorial field”—as highlighted by Nick (2007)—might be responsible for the direction-dependent stability of MTs.

Although this process did not appear to involve the AFs, there still is considerable amount of evidence from pharmacological and genetic studies that rearrangements of the MTs and AFs often depend on one another—for excellent reviews on this matter see Nick (2007) and Collings (2008). One recent contribution to that view was provided by Sampathkumar et al. (2011) who studied the structural association between AFs and MTs using live-cell microscopy. The authors observed that the re-assembly of AFs after drug-induced depolymerization was dependent on MTs. In particular, short AFs initially appeared colocalized with MTs and were motile along the MTs. Similarly, MTs also required an intact AF network to recover. This study indicated that cytoskeletal cross-talk might be enhanced during major cytoskeletal rearrangements as typically present under harsh environmental or stress conditions. Another inspiring point made by the authors was that MT-based molecular motors might be involved in the nucleation, positioning or transportation of AFs. This observation together with other recent studies provide interesting evidences for plant-specific cross-talk between the AFs and MTs—a scenario that has gained attention in the community, and some promising candidates that may contribute to the interdependence between MTs and AFs have also emerged. In this review we will summarize our understanding for one of these candidates, namely the plant-specific actin-binding kinesin-14 motor proteins (KCHs).

## The Plant-specific Actin-binding Kinesin-14 Motor Proteins

The model organism *Arabidopsis thaliana* is highly unusual in terms of its rich pool of MT-based kinesin motor proteins. Among all so-far sequenced organisms *Arabidopsis* features one of the largest sets of kinesins (61 compared to 52 in poplar, 41 and 45 in two cultivars of rice, and 45 in humans), surprisingly only beaten by *Physcomitrella* with a set of 71 kinesins (Reddy and Day, 2001; Richardson et al., 2006).These large sets are mainly due to the expansion of the kinesin-7 and kinesin-14 families (15 and 21 members in *Arabidopsis*, respectively). Kinesin-14 motors are an outstanding group among kinesins because of their minus-end directed motility (Cross, 2009). The direction of motility is inferred from the position of the motor domain within the amino acid sequence of the motor: kinesins with N-terminal motor domains typically move toward the plus-end of MTs whereas the ones with C-terminal motor domains move toward the minus-end (Hirokawa et al., 2009). However, only 5 of the 21 kinesin-14′s in *Arabidopsis* have their motor domain at the C-terminus (Reddy and Day, 2001). Still, the remaining kinesin-14s (11 with internal and 5 with N-terminal motor domains) are predicted to be minus-end directed due to the presence of the consensus neck linker motif found among kinesins that move toward the minus-end (Endow, 1999). This neck linker connects the motor to a coiled-coil dimerization domain and is responsible for the bias of the motor heads into one direction.

It has been proposed that land plants (i.e., embryophytes) evolved novel minus-end directed kinesins in order to compensate for the loss of cytoplasmic dynein (Lawrence et al., 2001; Wickstead and Gull, 2007). Dyneins are motor proteins responsible for intraflagellar transport, organization of the Golgi apparatus and spindle poles, and for moving nuclei, vesicles and chromosomes in animals, fungi, algae and mosses. In this context, it is interesting to note that the large set of kinesins of the moss *Physcomitrella* suggests that the expansion of the kinesin family perhaps occurred in parallel to the loss of cytoplasmic dynein. Moreover, the kinesin expansion, particularly that of the kinesin-7 and -14 subgroups, has been dated to the emergence of flowering plants (Richardson et al., 2006). To achieve fertilization of the female gamete flowering plants utilize polar pollen tube growth instead of cilia-driven spermatozoids. This evolutionary change may have caused the loss of the axonemal dyneins, which drive the collective beating of cilia. It is therefore tempting to speculate that part of the kinesin-7′s and -14′s might function in anisotropic cell expansion, as necessary for elongated pollen tubes.

**FIGURE 1:**
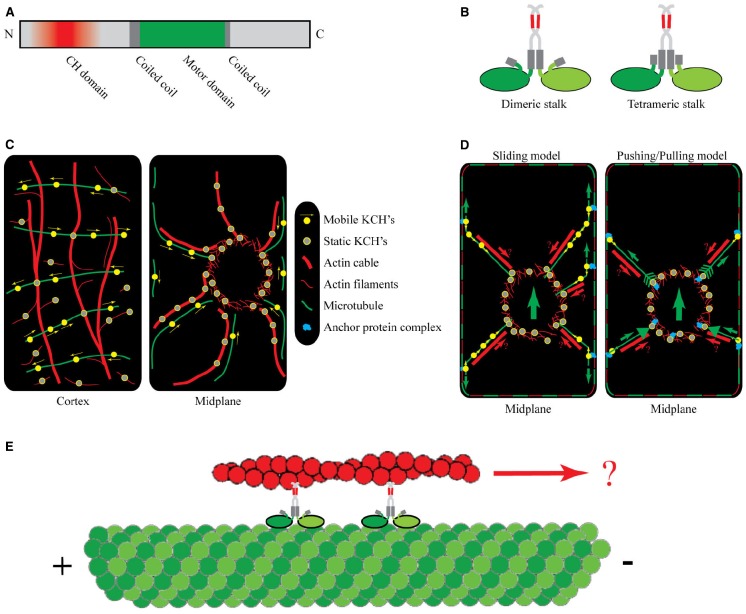
**Protein structure, intracellular localization, and putative function of KCHs. (A)** Representative domain structure of KCHs. The N-terminal CH domain (red) is necessary but not sufficient for actin binding. The motor domain (green) contains the ATP and MT binding sites. It is situated between coiled-coil domains, which facilitate dimerization. **(B)** Schematic of two possible conformations of the KCH dimer with the MT- and AF-binding sites being freely accessible: a flexible dimeric stalk (left) and a stiffer tetrameric stalk configuration (right). **(C)** Intracellular localization of KCHs at the cell cortex (left) and in the cell midplane (right) of an idealized BY2 cell during interphase. **(D)** Two alternative working models of KCH functioning in pre-mitotic nuclear positioning: “*Sliding model*” (left) and “*Pushing/pulling model*” (right). The cortical cytoskeleton is depicted as dashed red-green frame. The small green arrows represent forces transmitted via MTs. The large green arrows represents the direction of the resulting net force. The red arrows (labeled with a question mark) indicate a speculative mechanism of force transmission via AFs. **(E)** One putative function of KCHs may be to transport AFs relative to MTs toward the minus-end.

Phylogenetic analyses of the kinesin-14 motors in *Arabidopsis* revealed that they cluster into distinct subgroups (Reddy and Day, 2001; Richardson et al., 2006). One of the subgroups contains seven members and is characterized by an internal motor domain flanked by coil–coils and a C-terminal calponin homology (CH) domain (see Figure 1A). Proteins that contain individual CH domains may be involved in signal transduction processes and cannot bind to AFs (Gimona and Mital, 1998), whereas proteins with CH domains in tandem typically associate with AFs, e.g., multiple CH domains provide FIMBRIN with its specificity for AFs (Gimona et al., 2002). Kinesin’s dimerization via coiled-coils most likely brings CH domains in sufficiently close contact to generate high affinity to AFs (see Figure 1B). In fact, except for the supposedly monomeric KatD (Tamura et al., 1999), and KP1 for which actin-binding was not tested (Ni et al., 2005), all examined KCHs show AF-binding potential.

While the binding potential of the KCHs to AFs is clear, the functions of KCHs *in vivo* are largely unexplored. Thus, tissue- and co-expression databases may help to predict in which cellular processes KCHs participate, and with which partner proteins KCHs cooperate. According to microarray-based gene expression databases, KCHs can be arranged into two groups: Genes in one group, containing *KatD*, *KinG*, *AT5G41310*, *AT3G10310*, and *AT1G09170*, show high expression in mature pollen and flower tissue or in the shoot apex (Table 1). These motors might therefore function during gametophyte and/or floral development. Interestingly, three of those genes are co-expressed with *EB1*, a MT-end tracking protein involved in the organization of MTs in the cortex and during polar growth (Chan et al., 2003; Galva et al., 2014). EB1 is thought to act as an integrator of protein complex assembly, allowing MT ends to anchor to membranes and AFs (Mathur et al., 2003; Sieberer et al., 2005). It is plausible that the KCHs might aid in the process of linking MT ends to AFs, and/or to transmit forces between MTs and AFs. Genes in a second group, containing *KP1* and *AT2G47500*, show high expression in phloem and xylem cells, respectively, in stems and hypocotyls or in siliques (Table 1). *AT2G47500* is co-expressed with *MIDD1*, encoding a MT-destabilizing protein necessary for the formation of pitted secondary cell walls (Oda and Fukuda, 2012). Interestingly, *AT2G47500* is also co-expressed with *MAP65-8*, and both genes were found as downstream targets of the VND7 transcription factor, a master regulator of xylem vessel formation (Yamaguchi et al., 2011). Based on these observations, we speculate that KP1 and AT2G47500 function during vascular tissue development and/or secondary cell wall formation.

**TABLE 1:** **Overview of tissue- and co-expression of the seven *Arabidopsis* KCHs**.

**AGI code**	**Other names**	**Co-expressed with^a^**	**Expressed in^b^**	**References**
*AT5G27000*	*KatD*	*EB1*	Mature pollen, flower stage	Tamura et al. (1999)
*AT1G63640*	*KinG*	*CHR17, CHR20, EB1, VILLIN1, PDI12, FANCM, SERK1*	Mature pollen	Buschmann et al. (2010)
*AT5G41310*	–	*CROLIN1*	Mature pollen flower stage	–
*AT3G10310*	–	*IQD8, KIN5B, PAKRP2, EB1C, MAP65-3*	Shoot apex flower stage	–
*AT1G09170*	–	*ESE2, TBL19, CAP1, BSK9, RHS6*	Mature pollen flower stage	–
*AT3G44730*	*KP1, KIN14H*	–	Stem (Phloem), Hypocotyl, Siliques	Ni et al. (2005) and Yang et al. (2011)
*AT2G47500*	–	*MAP65-8, MYOB3, MIDD1, NET1C*	Stem (Xylem)	–

^a^Co-expression networks were obtained using PlaNet (Mutwil et al., 2011). ^b^Tissue expression levels were analyzed using the eFP browser (Winter et al., 2007).

It’s interesting to note that three KCHs from both groups (*AT1G63640*, *AT5G41310*, and *AT2G47500*) are co-expressed with actin-binding proteins, such as *MYOB3*, *VILLIN1*, and *CROLIN* (Khurana et al., 2010; Jia et al., 2013), perhaps suggesting potential partner proteins that could help in cross-linking the cytoskeleton.

## Functional Role of the KCHs

Although a detailed picture of the role of the KCHs in *Arabidopsis* is still missing, considerable insight has been obtained from studies of KCH-homologs in cotton (*Gossypium hirsutum*), rice (*Oryza sativa*), and tobacco (*Nicotiana tabacum*). The *in vivo* colocalization of KCHs with MTs and AFs was first shown by Preuss et al. (2004) for KCH1 in cotton cytoplasts, and later also for the cotton KCH2, rice KCH1 (identical to rice kinesin O12; Umezu et al., 2012), the *Arabidopsis* KinG, and the tobacco KCH1 (Xu et al., 2007, 2009; Buschmann et al., 2010; Frey et al., 2010, 2009; Umezu et al., 2010; Klotz and Nick, 2011). KCHs were shown to also bind to AFs and MTs *in vitro*. Interestingly, Xu et al. (2009) reported that KCH2 from cotton not only bundled MTs and AFs, but it also cross-linked them *in vitro*. These data indicated that KCHs might be capable of binding both arrays at the same time. It is still unclear, however, if KCHs can transport AFs along MTs. Indication that this might not be the case was provided by Umezu et al. (2010), who observed that AFs inhibited the ATPase activity of OsKCH1 in the presence of MTs. However, an alternative interpretation of this study could be that OsKCH1 binds more strongly to AFs compared to MTs. Thus, AFs might sequester KCHs from the solution leading to fewer free motors available for landing and walking on MTs thus decreasing the overall ATPase activity. In fact, Xu et al. (2009) already noticed that AFs bundled more effectively as compared to MTs at the same concentration of KCH2.

The existing *in vitro* data on the AF-binding potential of KCHs gained further support by *in vivo* studies of their intracellular localization. These studies, performed mostly in BY2 cells, showed that during interphase KCHs colocalized with cortical and radial MTs, often in transversely oriented strings of dots (Figure 1C, left panel, Preuss et al., 2004; Frey et al., 2009, 2010; Xu et al., 2009; Klotz and Nick, 2011). More interesting, however, was the finding that KCHs decorated several AF structures. Colocalization was found with AFs in the perinuclear region and with radial actin cables emanating out from the perinuclear region toward the cell periphery (Figure 1C, right panel, Frey et al., 2009; Klotz and Nick, 2011). There exist conflicting observations on the binding potential of KCHs to AFs at the cell cortex. On the one hand, Preuss et al. (2004) showed that GhKCH1 decorated fine, transversely oriented AFs at the cortex and Frey et al. (2009) showed that OsKCH1 was found to localize to crossovers of cortical MTs and AFs. On the other hand, KCHs might exclusively bind to cortical MTs based on two observations. First, treatment with oryzalin, a MT-depolymerizing drug, caused the cortical signal of KCHs to be lost whereas the decoration of the perinuclear region and radial actin cables remained unaltered (Frey et al., 2009; Klotz and Nick, 2011). Conversely, disruption of the AFs by treatment with Latrunculin B left the cortical signal of KCHs unaltered whereas the decoration of the perinuclear region and actin cables became diffuse. Second, using live-cell imaging to investigate the movement of KCHs on cortical MTs, Klotz and Nick (2011) found that NtKCH moved toward the minus-ends of cortical MTs being uncoupled from AFs. These observations imply that the cross-linking potential of KCHs might not be important for its function at the cell cortex.

In fact, there is evidence that KCHs might have an additional function during mitosis, because the localization and expression patterns of KCHs are cell-cycle dependent, i.e., the levels of KCH transcripts were shown to be low during interphase but elevated during mitosis (Klotz and Nick, 2011). In addition, the localization pattern also changed: upon onset of mitosis, KCH relocated from the cortex to decorate radial actin cables, and was found at two poles around the perinuclear region. This indicates that the actin-binding potential of KCHs might be more relevant during mitosis than during interphase. This relocation of KCHs caused Frey et al. (2010) to speculate that KCHs have dual functions; one during interphase and one during mitosis. In fact, this hypothesis is consistent with mutant studies in rice. There, *kch1* insertion (knock-down) mutants displayed impaired cell elongation as compared to wild-type, a defect that was partially compensated for by an increase in cell division in rice coleoptiles (Frey et al., 2010). Overexpression of KCH1, on the other hand, led to longer coleoptiles and delayed pre-mitotic nuclear migration. Thus, KCH1 seems to be involved in two processes: cell elongation and cell division.

GhKCH1 and GhKCH2 are proposed to play active roles during cell elongation (Preuss et al., 2004; Xu et al., 2009), because both are highly expressed in elongating cotton fibers. Taken together with the localization of the rice and tobacco KCHs to cortical MTs, this indicates that KCHs can stabilize transverse MT arrays at the cell cortex during interphase and thus promote polar cell expansion. It is not clear, however, if KCHs need the AF-cross-linking capability to perform this function. The involvement of OsKCH1 in cell division is not surprising because *OsKCH1* is highly expressed in tissues with meristematic activity, such as young roots, young leaves and young flowers (Frey et al., 2010). Interestingly, in the rice knock-down mutant *kch1*, no morphological changes of the spindle body were detected. Instead KCHs seem to be involved in slowing down nuclear positioning prior to mitosis.

Two models were proposed by Frey et al. (2010) to describe the function of KCHs in nuclear positioning. In the “*sliding model*” (see Figure 1D, left panel), static KCHs are bound to perinuclear AFs and anchor the minus-ends of radial MTs. Together with peripheral AFs running in parallel, the radial MTs tether to the cell cortex, either directly via KCHs or via a yet to be determined anchor protein complex. The cortex-tethered KCHs can bind to the plus-ends of radial MTs and walk toward their minus-ends, thereby generating a pulling force onto the nucleus by sliding MTs along the cortex. In an alternative “*pushing/pulling model*” (see Figure 1D, right panel), the force could originate from the dynamic instability of MTs. Here, the ends of radial MTs are stably anchored to the nuclear envelope and the cell cortex via anchoring protein complexes that contain KCH. The anchoring is thought to leave the growing and shrinking dynamics of MTs unaltered, so that growing MTs would generate inward-directed “pushing” forces, whereas shrinking MTs would generate outward-directed “pulling” forces.

For these two models to work, an asymmetry is required: for the sliding model, either the number of radial MTs or the number of pulling motors need to be asymmetric in order to generate a net force toward one side. Likewise, for the pushing/pulling model either the number of radial MTs or their dynamics, i.e., growing and shrinking rates, need to be asymmetric. It is not clear, whether KCHs are distributed in such an asymmetric fashion.

Furthermore, both models do not explain why the knock-out and the overexpression of KCHs lead to pre-mitotic migration being sped up or slowed down, respectively. Both models would predict quite the opposite. This discrepancy could be explained if nuclear positioning would be steered by two oppositely directed force-feedback mechanisms. In such a scenario, KCHs could take over the job of slowing down nuclear movement by working against the force generated by another process. It is tempting to speculate that KCHs themselves could in part contribute to this opposing force. For example, KCHs walking on radial MTs toward the nucleus could bind to radial actin cables running in parallel and thus transmit pushing forces onto the nucleus. Such a function would be in accordance with experimental data: KCHs were found to localize to radial actin cables and KCHs possess AF-cross-linking capability. If that scenario bears some validity it should be possible to directly observe individual KCHs transporting AFs along MTs both *in vitro* and *in vivo*.

Moreover, it could also be envisioned that the KCHs stabilize the interphase cytoskeletal arrays, e.g., by providing the cytoskeleton with an internal resistance against reorganization, thereby preventing nuclear movement. Overexpression of KCH would thus make the cytoskeleton more resistant leading to a delay in nuclear migration. The knock-out of KCH would in turn make the cytoskeleton susceptible to reorganizations leading to an earlier onset of nuclear migration. However, experimental evidence for such a scenario is lacking.

At first glance, the proposed model of nuclear positioning by Frey et al. (2010) is of striking analogy to models proposed for the yeasts *Saccharomyces cerevisiae* and *Schizosaccharomyces pombe* (Adames and Cooper, 2000), but also for *Caenorhabditis elegans*, *Drosophila melanogaster* (Sharp et al., 2000; Dujardin and Vallee, 2002), and even for motile fibroblasts of mouse (Levy and Holzbaur, 2008). In yeast, two alternative mechanisms govern the nuclear movement and the partitioning of the spindle between the mother cell and the newly formed bud (Adames and Cooper, 2000). The first mechanism relies on anchoring of nuclear MTs to the bud cortex and subsequent depolymerization leading to a pulling force. The second mechanism relies on dynein/dynactin-dependent sliding of spindle-MTs along the cell cortex into the bud. The model put forth by Levy and Holzbaur (2008) on the positioning of the nucleus during forward progression of motile fibroblasts represents another striking similarity to the model of Frey et al. (2010). The authors suggested that dynein has a dual-function in cell migration: one function being the tethering of MT plus-ends to the cell cortex allowing the centrosomes to be held in place at the cell center, the other function involves localization of dynein to the nuclear envelope where it exerts forces onto perinuclear MTs—and thus the nucleus—to maintain nuclear centrality during translocation.

In light of these similarities, it is tempting to speculate once more that KCHs might represent the functional plant homologs for cytoplasmic dynein. Shedding light onto this attractive analogy will certainly boost our understanding of the evolution of embryophytes. In particular, why do land plants need a large cassette of KCHs instead of dyneins to grow and develop?

## Outlook

*In vitro* reconstitution assays, in which the motor can be studied in controlled environments, will be key to understanding how KCH motors mediate cross-talk between AFs and MTs (Dixit, 2012). One putative function of KCHs, namely the transportation of AFs along MTs observed by Sampathkumar et al. (2011), has never been demonstrated *in vitro* (see illustration in Figure 1E). Furthermore, the sliding and the pushing/pulling model anticipate that KCHs can capture the minus-ends of MTs and attach these to the nuclear envelope. To fulfill this function, KCHs need to stably adhere to the MT ends; either via an adherence mechanism to the MT minus-end when the MTs depolymerize or via a minus-end tracking protein association. Moreover, the polarity of the MTs determines the direction in which KCHs move. Does the polarity of AFs in turn also control motor properties? Finally, how strong are the associations of KCHs with AFs and MTs? In other words, how much force can individual motors withstand before being detached? How is the switch between mobile KCHs and static KCHs regulated? Answering such questions would represent a great leap forward for the cell biology of plant motors.

While such *in vitro* assays would give important insights into the function of KCHs, it is also evident that more research *in planta* is required. While previous studies mainly focused on the economically relevant crop species cotton and tobacco, most of the intracellular work was done in cultured BY2 cells. Although this system is perfectly suited to investigate colocalization of KCHs with AFs and MTs, it is ignoring the fact that *KCH* expression is tissue-dependent, and it is difficult to infer results from cell suspensions to a growing plant. Instead, studying the function of KCHs in the tissue in which they are expressed strongest offers great potential to shed light on their physiological role. Naturally, *Arabidopsis* resembles a promising organism to focus on due to its large set of described mutations and functional predictions of proteins (SALK insertion mutants are available for all 7 KCHs). However, so far only three of the seven *Arabidopsis* KCHs have been reported on, and thus a lot remains to be investigated. With the help of cutting-edge imaging technologies, such as live-cell and super-resolution microscopy, big data proteomics as well as species-wide genome analyses, we expect major advances during the coming years.

### Conflict of Interest Statement

The authors declare that the research was conducted in the absence of any commercial or financial relationships that could be construed as a potential conflict of interest.
